# Amniotic Fluid and Placental Membranes as Sources of Stem Cells: Progress and Challenges 2.0

**DOI:** 10.3390/ijms242216020

**Published:** 2023-11-07

**Authors:** Tullia Maraldi, Valentina Russo

**Affiliations:** 1 Department of Biomedical, Metabolic and Neural Sciences, University of Modena and Reggio Emilia, Via del Pozzo 71, 41125 Modena, Italy; 2 Faculty of Bioscience and Agro-Food and Environmental Technology, Agriculture and Environment, University of Teramo, Via Renato Balzarini 1, 64100 Teramo, Italy

The aim of the second edition of this Special Issue was to collect both review and original research articles that investigate and elucidate the possible therapeutic role of perinatal stem cells in pathological conditions, such as cardiovascular and metabolic diseases, as well as inflammatory, autoimmune, musculoskeletal, and degenerative diseases.

Since mounting evidence has demonstrated the efficacy of different sources of stem cells [1,2,3,4] and the derived secretome [5,6,7,8], in recent years, perinatal stem cells have been considered as an alternative and available cell source for biomedical research in vitro [9,10] and in vivo [11], including clinical trials [12,13,14].

For example, amniotic membrane (epithelial and mesenchymal) and amniotic fluid stem cells possess many advantages. Unlike embryonic cells, amniotic cells are free from tumorigenicity and ethical considerations, since these cells can be extracted from discarded fetal material [15]. Moreover, they possess embryonic stem-cell-like differentiation capabilities [16], and, similar to mesenchymal stem cells, they are also able to modulate local immune responses [17], allowing their use in allo- and xeno-transplantation settings [18,19,20].

These properties, among others, make amniotic cells attractive for cellular therapy; however, translating laboratory findings into clinical practice still requires a lot of effort [21]. Here, we summarize the main evidence collected in this Special Issue, such as suggestions on culture methods and findings on the amniotic cell mechanisms of action in counteracting inflammatory-based and degenerative diseases.

The results obtained from several clinical trials for the treatment of many disorders are controversial [22]. Therefore, there is a need to improve MSC culture/production in order to enhance their therapeutic properties. The first idea that can be deduced by two original research papers is that three-dimensional (3D) culturing techniques can modify and even ameliorate the properties of amniotic cells, since they enable a better representation of in vivo conditions, increasing cell-to-cell interactions [23]. Indeed, despite the widely excepted benefits of 3D cell culturing, research on three-dimensional hAFSC cultures is very limited and mainly involves an analysis of cells grown on transplantable scaffolds [24]. In this Special Issue, with different approaches, such as RNA-seq, qRT-PCR, methylome analysis, and an investigation of secreted factors, Gallo et al. [25] demonstrated that culturing human amnion-derived MSCs (hAMSCs) as spheroids can improve proliferation/differentiation, as well as immunomodulatory and angiogenic processes.

Similarly, Valiuliené et al. [26] showed that this strategy has efficacy in upregulating the expression of pluripotency genes, NF-κB−TNFα pathway genes, and associated miRNAs (miR103a-5p, miR199a-3p, and miR223-3p) in human amniotic fluid stem cells (hAFSCs). Moreover, they investigated the neurogenic properties of hAFSCs when grown in 3D cultures. The neural differentiation of hAFSC spheroids increased the expression of SOX2, miR223-3p, and MSI1, as well as neural differentiation-associated gene expression levels, in comparison to 2D-treated cultures.

hAFSCs, as a type of MSC, play a significant therapeutic role in animal models of in-flammation-related diseases, demonstrating their anti-inflammatory effects [27]. However, the effect of hAFSCs on acquired immunity in vivo, especially on regulatory T cells, has not yet been fully elucidated. In particular, the relationship between hAFSCs and Tregs in vivo has not been investigated, although an in vitro study showed that hAFSCs increased Tregs [28]. Abe et al. [29] showed that hAFSCs ameliorated the thioglycollate-induced inflammation, a peritonitis mice model, by forming aggregates with host immune cells, such as macrophages, T cells, and B cells in the peritoneal cavity. Furthermore, the number of regulatory T cells increased in the peritoneal cavity. We can assume that, in addition to helping innate immunity, hAFSCs could also aid the acquired immune system in vivo against inflammation-related diseases by increasing regulatory T cells.

Even in the case of another type of inflammation, such as persistent post-breeding-induced endometritis (PPBIE), in this Special Issue, amniotic cells were proven to be effective in modulating inflammation by applying extracellular vesicles derived from amniotic mesenchymal stromal cells (AMSC-EVs) in stallion semen. To date, only one study has evaluated the in vivo effects of MSC-CM intrauterine infusion on mares that are susceptible to PPBIE [30], and another study showed that AMSC-conditioned medium (AMSC-CM) was effective in the replenishment of endometrial cells and uterine regeneration [31]. Lange-Consiglio and colleagues [32] demonstrated that the supplementation of AMSC-EVs to semen resulted in the successful modulation of post-insemination inflammatory responses in mares that could be able to prevent PPBIE.

Neuroinflammation is involved in neuronal cell death that occurs in neurodegenerative diseases such as Alzheimer’s disease (AD) [33]. Microglia play important roles in regulating the brain amyloid beta (Aβ) levels [34], and, in the study by Zavatti et al. [35], the effect of exosomes derived from human amniotic fluid stem cells (hAFSCs-exo) on activated BV-2 microglia cells was evaluated using lipopolysaccharide (LPS) as the neuroinflammation model. They demonstrated that the activation of pro-inflammatory microglia was prevented when exposed to hAFSC-exo, while the increases in oxidative stress and apoptosis occurring in neurons in the presence of both microglia and Aβ were significantly inhibited. They concluded that hAFSC-exo mitigated an inflammatory injury caused by microglia and significantly counteracted the neurotoxicity, supporting the idea that hAFSC-exo can be a potential therapeutic agent for inflammation-related neurological conditions.

Although all this promising evidence on amniotic-derived stem cells presents an attractive tool for regenerative medicine and cell-based therapy, they could act as a reservoir of persistent viruses by increasing the risk of failure of stem-cell-based therapies and viral transmission, especially in immunocompromised patients. Indeed, MSCs have been demonstrated to be susceptible to infection by a variety of viruses that represent prominent pathogens in immunocompromised hosts, including HSV-1, VZV, and CMV [36,37]. This phenomenon was demonstrated by Bua et al. [38] for parvovirus B19V (B19V), which is a common human pathogen that infects bone marrow erythroid progenitor cells, leading to transient or persistent anemia. Indeed, this virus is able to cross the placenta, infecting the fetus, and persists in several tissues. Even if cells derived from the fetal membrane (FM-MSCs) were not able to support viral replication, they can be infected by B19V and allow persistence over time in the infected cultures.

All in all, multiple drawbacks are associated with autologous sources, including donorsite morbidity, a dearth of studies, and variability in both patient-reported and clinical/functional outcomes. On the other hand, allogenic sources address several of these concerns, and continue to be a suitable source of mesenchymal stem cells (MSCs), such as amniotic suspension allograft, amniotic membrane, and amniotic fluid [39].

In conclusion, since stem cell use represents an unprecedented strategy for several auto- and allo-therapies but product availability and mass production remain challenges [40], future studies are needed to explain the potential role of amniotic stem cells in cell therapy, in particular to find the best protocols to manipulate cells, obtain secretome, and apply cell-derived treatment, still maintaining the safety of the host in the various procedures.

## References

[1] Xin Q., Zhu W., He C., Liu T., Wang H. (2023). The effect of different sources of mesenchymal stem cells on microglia states. Front. Aging Neurosci..

[2] Prajwal G.S., Jeyaraman N., Kanth V.K., Jeyaraman M., Muthu S., Rajendran S.N.S., Rajendran R.L., Khanna M., Oh E.J., Choi K.Y. (2022). Lineage Differentiation Potential of Different Sources of Mesenchymal Stem Cells for Osteoarthritis Knee. Pharmaceuticals.

[3] Kouchakian M.R., Baghban N., Moniri S.F., Baghban M., Bakhshalizadeh S., Najafzadeh V., Safaei Z., Izanlou S., Khoradmehr A., Nabipour I. (2021). The Clinical Trials of Mesenchymal Stromal Cells Therapy. Stem Cells Int..

[4] Jahani S., Zare N., Mirzaei Y., Arefnezhad R., Zarei H., Goleij P., Bagheri N. (2023). Mesenchymal stem cells and ovarian cancer: Is there promising news?. J. Cell. Biochem..

[5] Yuan W., Wu Y., Huang M., Zhou X., Liu J., Yi Y., Wang J., Liu J. (2022). A new frontier in temporomandibular joint osteoarthritis treatment: Exosome-based therapeutic strategy. Front. Bioeng. Biotechnol..

[6] Xia Y., Yang R., Hou Y., Wang H., Li Y., Zhu J., Fu C. (2022). Application of mesenchymal stem cell-derived exosomes from different sources in intervertebral disc degeneration. Front. Bioeng. Biotechnol..

[7] Mattoli S., Schmidt M. (2023). Investigational Use of Mesenchymal Stem/Stromal Cells and Their Secretome as Add-On Therapy in Severe Respiratory Virus Infections: Challenges and Perspectives. Adv. Ther..

[8] Sanie-Jahromi F., Mahmoudi A., Khalili M.R., Nowroozzadeh M.H. (2022). A Review on the Application of Stem Cell Secretome in the Protection and Regeneration of Retinal Ganglion Cells; a Clinical Prospect in the Treatment of Optic Neuropathies. Curr. Eye Res..

[9] Witkowska-Zimny M., Wrobel E. (2011). Perinatal sources of mesenchymal stem cells: Wharton’s jelly, amnion and chorion. Cell. Mol. Biol. Lett..

[10] Hu Z., Luo Y., Ni R., Hu Y., Yang F., Du T., Zhu Y. (2023). Biological importance of human amniotic membrane in tissue engineering and regenerative medicine. Mater. Today Bio.

[11] Pichlsberger M., Jerman U.D., Obradović H., Tratnjek L., Macedo A.S., Mendes F., Fonte P., Hoegler A., Sundl M., Fuchs J. (2021). Systematic Review of the Application of Perinatal Derivatives in Animal Models on Cutaneous Wound Healing. Front. Bioeng. Biotechnol..

[12] Russo E., Alberti G., Corrao S., Borlongan C.V., Miceli V., Conaldi P.G., Di Gaudio F., La Rocca G. (2023). The Truth Is Out There: Biological Features and Clinical Indications of Extracellular Vesicles from Human Perinatal Stem Cells. Cells.

[13] Pfister P., Wendel-Garcia P.D., Meneau I., Vasella M., Watson J.A., Bühler P., Rittirsch D., Lindenblatt N., Kim B.S. (2023). Human amniotic membranes as an allogenic biological dressing for the treatment of burn wounds: Protocol for a randomized-controlled study. Contemp. Clin. Trials Commun..

[14] Fernández-Garza L.E., Barrera-Barrera S.A., Barrera-Saldaña H.A. (2023). Mesenchymal Stem Cell Therapies Approved by Regulatory Agencies around the World. Pharmaceuticals.

[15] Joerger-Messerli M.S., Marx C., Oppliger B., Mueller M., Surbek D.V., Schoeberlein A. (2016). Mesenchymal Stem Cells from Wharton’s Jelly and Amniotic Fluid. Best Pract. Res. Clin. Obstet. Gynaecol..

[16] Zhang Q., Lai D. (2020). Application of human amniotic epithelial cells in regenerative medicine: A systematic review. Stem Cell Res. Ther..

[17] Yang C., Wu M., You M., Chen Y., Luo M., Chen Q. (2021). The therapeutic applications of mesenchymal stromal cells from human perinatal tissues in autoimmune diseases. Stem Cell Res. Ther..

[18] Wassmer C.H., Berishvili E. (2020). Immunomodulatory Properties of Amniotic Membrane Derivatives and Their Potential in Regenerative Medicine. Curr. Diab. Rep..

[19] Papait A., Silini A.R., Gazouli M., Malvicini R., Muraca M., O’Driscoll L., Pacienza N., Toh W.S., Yannarelli G., Ponsaerts P. (2022). Perinatal derivatives: How to best validate their immunomodulatory functions. Front. Bioeng. Biotechnol..

[20] Zafar A., Lee J., Yesmin S., Paget M.B., Bailey C.J., Murray H.E., Downing R. (2019). Rotational culture and integration with amniotic stem cells reduce porcine islet immunoreactivity in vitro and slow xeno-rejection in a murine model of islet transplantation. Xenotransplantation.

[21] Pozzobon M., D’Agostino S., Roubelakis M.G., Cargnoni A., Gramignoli R., Wolbank S., Gindraux F., Bollini S., Kerdjoudj H., Fenelon M. (2022). General consensus on multimodal functions and validation analysis of perinatal derivatives for regenerative medicine applications. Front. Bioeng. Biotechnol..

[22] Lukomska B., Stanaszek L., Zuba-Surma E., Legosz P., Sarzynska S., Drela K. (2019). Challenges and Controversies in Human Mesenchymal Stem Cell Therapy. Stem Cells Int..

[23] Kouroupis D., Correa D. (2021). Increased Mesenchymal Stem Cell Functionalization in Three-Dimensional Manufacturing Settings for Enhanced Therapeutic Applications. Front. Bioeng. Biotechnol..

[24] Mohammed E.E.A., Beherei H., El-Zawahry M., Farrag A.R., Kholoussi N., Helwa I., Gaber K., Allam M.A., Mabrouk M., Aleem A.K.A. (2019). Combination of Human Amniotic Fluid Derived-Mesenchymal Stem Cells and Nano-hydroxyapatite Scaffold Enhances Bone Regeneration. Open Access Maced. J. Med. Sci..

[25] Gallo A., Cuscino N., Contino F., Bulati M., Pampalone M., Amico G., Zito G., Carcione C., Centi C., Bertani A. (2022). Changes in the Transcriptome Profiles of Human Amnion-Derived Mesenchymal Stromal/Stem Cells Induced by Three-Dimensional Culture: A Potential Priming Strategy to Improve Their Properties. Int. J. Mol. Sci..

[26] Valiulienė G., Zentelytė A., Beržanskytė E., Navakauskienė R. (2023). Effect of 3D Spheroid Culturing on NF-κB Signaling Pathway and Neurogenic Potential in Human Amniotic Fluid Stem Cells. Int. J. Mol. Sci..

[27] Ochiai D., Abe Y., Fukutake M., Sato Y., Ikenoue S., Kasuga Y., Masuda H., Tanaka M. (2021). Cell sheets using human amniotic fluid stem cells reduce tissue fibrosis in murine full-thickness skin wounds. Tissue Cell..

[28] Mareschi K., Castiglia S., Sanavio F., Rustichelli D., Muraro M., Defedele D., Bergallo M., Fagioli F. (2016). Immunoregulatory effects on T lymphocytes by human mesenchymal stromal cells isolated from bone marrow, amniotic fluid, and placenta. Exp. Hematol..

[29] Abe Y., Ochiai D., Taguchi M., Kanzaki S., Ikenoue S., Kasuga Y., Tanaka M. (2022). Human Amniotic Fluid Stem Cells Ameliorate Thioglycollate-Induced Peritonitis by Increasing Tregs in Mice. Int. J. Mol. Sci..

[30] de Oliveira Tongu E.A., Segabinazzi L.G.T.M., Alvarenga M.L., Monteiro A., Papa F.O., Alvarenga M.A. (2021). Allogenic Mesenchymal Stem Cell-Conditioned Medium Does Not Affect Sperm Parameters and Mitigates Early Endometrial Inflammatory Responses in Mares. Theriogenology.

[31] Corradetti B., Correani A., Romaldini A., Marini M.G., Bizzaro D., Perrini C., Cremonesi F., Lange-Consiglio A. (2014). Amniotic Membrane-Derived Mesenchymal Cells and Their Conditioned Media: Potential Candidates for Uterine Regenerative Therapy in the Horse. PLoS ONE.

[32] Lange-Consiglio A., Gaspari G., Funghi F., Capra E., Cretich M., Frigerio R., Bosi G., Cremonesi F. (2023). Amniotic Mesenchymal-Derived Extracellular Vesicles and Their Role in the Prevention of Persistent Post-Breeding Induced Endometritis. Int. J. Mol. Sci..

[33] Singh A., Kukreti R., Saso L., Kukreti S. (2019). Oxidative stress: A key modulator in neurodegenerative diseases. Molecules.

[34] Butovsky O., Weiner H.L. (2018). Microglial signatures and their role in health and disease. Nat. Rev. Neurosci..

[35] Zavatti M., Gatti M., Beretti F., Palumbo C., Maraldi T. (2022). Exosomes Derived from Human Amniotic Fluid Mesenchymal Stem Cells Preserve Microglia and Neuron Cells from Aβ. Int. J. Mol. Sci..

[36] Avanzi S., Leoni V., Rotola A., Alviano F., Solimando L., Lanzoni G., Bonsi L., Di Luca D., Marchionni C., Alvisi G. (2013). Susceptibility of human placenta derived mesenchymal stromal/stem cells to human herpesviruses infection. PLoS ONE.

[37] Smirnov S.V., Harbacheuski R., Lewis-Antes A., Zhu H., Rameshwar P., Kotenko S.V. (2007). Bone-marrow-derived mesenchymal stem cells as a target for cytomegalovirus infection: Implications for hematopoiesis, self-renewal and differentiation potential. Virology.

[38] Bua G., Marrazzo P., Manaresi E., Gamberini C., Bonsi L., Alviano F., Gallinella G. (2023). Non-Permissive Parvovirus B19 Infection: A Reservoir and Questionable Safety Concern in Mesenchymal Stem Cells. Int. J. Mol. Sci..

[39] Aratikatla A., Maffulli N., Rodriguez H.C., Gupta M., Potty A.G., Gupta A. (2022). Allogenic Perinatal Tissue for Musculoskeletal Regenerative Medicine Applications: A Systematic Review. Biomedicines.

[40] Miatmoko A., Hariawan B.S., Cahyani D.M., Dewangga S.S., Handoko K.K., Purwati, Sahu R.K., Hariyadi D.M. (2023). Prospective use of amniotic mesenchymal stem cell metabolite products for tissue regeneration. J. Biol. Eng..

